# The Economic Cost of FAS

**Published:** 1994

**Authors:** Gregory Bloss

**Affiliations:** Gregory Bloss, M.A., is an economist in the Office of Policy Analysis, National Institute on Alcohol Abuse and Alcoholism, Rockville, Maryland

## Abstract

Estimates of the total cost of FAS are highly dependent on factors such as incidence rates for the disorder. Estimates of the lifetime costs involved with a single case of FAS may be more valuable.

Several studies (Abel and Sokol 1987, 1991*a*,*b*; Harwood and Napolitano 1985; Rice et al. 1990) have presented estimates of the economic cost to the Nation that results from fetal alcohol syndrome (FAS). Because of differences in the underlying assumptions employed in producing the estimates, the resulting costs cover a wide range. Much of this variation may be attributed to differences in the incidence rates (i.e., the number of FAS cases per 1,000 live births) on which the estimates are based. Further differences reflect discrepancies in the cost components, which are the specific adverse effects of FAS for which costs are computed, that are included in the studies. Estimates of the total economic cost that results from FAS provide a general idea of the dimensions of the problem, but they are of little help in designing or evaluating policies to counter the adverse effects of FAS. Estimates of the lifetime cost associated with a typical FAS case are more useful for such policy-related applications.

## Differences Among Cost Estimates of FAS

As mentioned above, a key source of differences among estimates of the total cost of FAS is variation in the FAS incidence rates used as the basis for cost calculations. Abel and Sokol (1987) estimated that in 1984, the cost of FAS in the United States was $321 million, based on an average incidence of 1.9 FAS cases per 1,000 live births. This incidence rate was an average drawn from several prospective and retrospective studies. (Prospective studies examined births that occurred while the study was in progress. Retrospective studies analyzed the records of previous births.) In a more recent paper, Abel and Sokol (199l*b*) produced a much lower cost estimate of $75 million based on an overall incidence rate of 0.33 FAS cases per 1,000 live births. This conservative estimate is derived entirely from prospective studies, which yield lower estimates of FAS incidence than do retrospective studies, in part because there are no prospective data for American Indians and other racial/ethnic groups that may face elevated risks of FAS.

Harwood and Napolitano (1985) generated a range of cost estimates using alternative FAS incidence rates of 1.0, 1.67, and 5.0 FAS cases per 1,000 live births. The last rate, which the researchers acknowledged was well above that suggested by the FAS literature, was provided for comparison as an upper boundary that might apply if children born with only some of the defects that constitute FAS were included in the analysis. This range of incidence rates accounted for the wide range of cost estimates reported by Harwood and Napolitano: from $1.95 billion to $9.69 billion.

The large discrepancies in the cost estimates obtained in various studies also are due in part to significant differences in the components of cost included in the calculations. All studies include the cost of care for FAS babies with low birth weight; the costs for surgical correction of FAS-related birth defects such as cleft palate, heart defects, and audiological defects; and the cost of care for those with moderate or severe mental retardation due to FAS. In arriving at their original estimate of $321 million, Abel and Sokol (1987) also included the cost of semi-independent supervised support for mildly retarded FAS patients age 21 and under, a cost category that was excluded from their later studies on the grounds that such care was generally required only after age 21 (none of the Abel and Sokol studies included costs beyond age 21). Rice and colleagues (1990) used the same approach and incidence rate as the original Abel and Sokol study but added costs of residential care for patients over 21. These costs account for 80 percent of their total estimate of $1.61 billion for 1985; the remaining 20 percent is accounted for by costs incurred through care of people with FAS-related birth defects and mental retardation. Harwood and Napolitano included estimates of the value of productivity lost as a result of FAS-related mental retardation as well as the cost of treatment and residential care for patients of all ages with FAS. Table 1 summarizes the cost estimates of the five studies.

## Limitations of Cost Estimates of FAS

In comparing these cost estimates, it is important to keep in mind two general limitations regarding their use and interpretation. First, some of the most significant aspects of the total burden of FAS are not measured in any of the studies, namely the pain and suffering (both physical and emotional) experienced by victims of FAS and their families. These effects are legitimately considered as costs, because people would willingly pay to avoid them. Nevertheless, cost-of-illness studies in general have omitted pain and suffering costs on the grounds that they are too difficult to estimate reliably (Rice et al. 1990; Hodgson and Meiners 1982).

Second, estimates of the total cost of FAS do not provide reliable guidance as to what policies for prevention and treatment of FAS are appropriate. Any specific FAS prevention or treatment policy must be evaluated on the basis of the costs and benefits associated with the specific policy, not the overall cost associated with FAS (Sindelar 1991; Wagstaff 1987). To conduct such evaluations, estimates of the cost associated with a single case of FAS are more helpful than are estimates of the total annual cost of all FAS cases in the Nation. For example, the economic benefit of a prevention policy may be interpreted as the cost that can be avoided by preventing FAS births. The benefit of a particular prevention policy can be calculated as the lifetime cost of a single FAS birth multiplied by the number of FAS cases that the policy is expected to prevent. This benefit can be compared with the cost of implementing the policy to determine whether the policy confers a net benefit, and how the benefits and costs of this policy compare with the benefits and costs associated with other policy approaches to the problem of FAS births.

## Estimating Lifetime Costs of FAS

The cost associated with an FAS birth is actually a stream of costs, spread unevenly over the life of the patient. To permit comparisons between a stream of costs and a one-time cost (such as might be associated with implementing a particular policy measure), analysts compute the *present discounted value* (PDV) of the stream of costs. In this calculation, costs that are incurred in the distant future are assigned smaller weights (discounted) relative to costs that are incurred nearer to the present. As a result, the PDV of a stream of future costs is less than the simple (undiscounted) sum of such costs. The degree to which future costs are discounted is determined by the *discount rate* selected by the analyst. The PDV of a given stream of costs can be interpreted as the amount of money that would have to be deposited in an interest-bearing account today (earning interest at a rate equal to the discount rate) so that the accumulated principal and interest would exactly defray the given stream of costs.^1^

Harwood and Napolitano (1985) have estimated the lifetime cost to age 65 associated with a typical case of FAS for a child born in 1980. The total (undiscounted) lifetime cost was estimated at $596,000 for each case of FAS, of which 68 percent represents direct expenditures necessary for treatment and residential care necessitated by FAS. The remaining 32 percent represents the value of FAS-related productivity losses. Using a discount rate of 6 percent, the PDV of the lifetime cost of a case of FAS was estimated at $163,000, of which 76 percent represents direct expenditures for treatment and residential care, and the remaining 24 percent represents productivity losses due to FAS.

In general, expanding efforts to prevent FAS will produce a net economic gain if the cost of preventing each additional case of FAS is less than the PDV of the lifetime cost of an FAS birth. Given the magnitude of the estimated PDV of the lifetime cost of an FAS case, one may conclude that major prevention efforts may be well justified on economic grounds.

## Figures and Tables

**Table 1:** Estimates of the Economic Cost of Fetal Alcohol Syndrome (FAS)

Study	Cost Components	FAS Incidence per 1,000 Live Births	Cost Estimate (millions)
Abel and Sokol (1987)	Treatment, care, and semi-independent support for age < 22	1.9	$321
Abel and Sokol (1991*a*)	Treatment and care for age < 22	1.9	$250
Abel and Sokol (1991*b*)	Treatment and care for age < 22	0.33	$75
Rice et al. (1990)^1^	Treatment and care for age < 22; residential care for all ages	1.9	$1,611
Harwood and Napolitano (1985)	Treatment, care, and lost productivity for all ages	1.0	$1,953
		1.67	$3,236
		5.0	$9,687

1 The Rice et al. (1990) estimate reported here is for 1985. The same study projected the estimated cost of FAS for 1988 at $1,837 million. Rice (1993) projected the cost of FAS for 1990 at $2,089 million.

## Footnotes

1 Note that a higher discount rate produces a lower PDV. Note also that PDV calculations are conceptually distinct from inflation adjustments, although the effects of inflation can be effectively removed from such calculations by appropriate choice of the discount rate.
